# Cortisol Testing in Septic Shock: An Evaluation of Diagnostic Performance and Predictors of Corticosteroid Use in a Middle Eastern Cohort

**DOI:** 10.3390/diagnostics15202588

**Published:** 2025-10-14

**Authors:** Fayez Alshamsi, Saeed Alkaabi, Maryam Nasser Mohamedali Alfadli, Naser Abdulla Naser Salem Alshkeili, Sultan Majed Ibrahim Alhosani, Adnan Agha

**Affiliations:** Department of Internal Medicine, College of Medicine and Health Sciences, United Arab Emirates University, Al Ain P.O. Box 15551, United Arab Emirates; f_ebrahim@uaeu.ac.ae (F.A.); 201602665@uaeu.ac.ae (S.A.); 201902315@uaeu.ac.ae (M.N.M.A.); 201900976@uaeu.ac.ae (N.A.N.S.A.); 201906788@uaeu.ac.ae (S.M.I.A.)

**Keywords:** critical illness-related corticosteroid insufficiency, septic shock, cortisol, intensive care, hydrocortisone, diagnostic accuracy, clinical utility, sepsis

## Abstract

**Background:** Critical illness-related corticosteroid insufficiency (CIRCI) diagnosis remains controversial, largely due to the complex pathophysiology of sepsis, which challenges the reliability of conventional biochemical testing. Recent international guidelines have moved away from strict cortisol threshold-based diagnostic criteria for adrenal insufficiency, relying more on clinical evaluation. However, the applicability and diagnostic accuracy of these revised approaches in non-Western populations remain unexplored. **Objective:** This study aimed to assessthe diagnostic accuracy and clinical utility of baseline total cortisol levels for guiding corticosteroid therapy in a cohort of patients admitted to the intensive care unit (ICU) with septic shock in a tertiary care hospital in the United Arab Emirates. **Methods:** A ten-year retrospective observational study (2012–2022) of intensive care patients with septic shock was conducted. CIRCI was operationally defined by a documented clinical decision to administer hydrocortisone >24 h. Receiver Operating Characteristic (ROC) analysis assessed diagnostic performance; Decision Curve Analysis (DCA) evaluated clinical utility of performed cortisol levels. **Results:** Among 43 patients in the ICU with septic shock, 13 (30.2%) received hydrocortisone (CIRCI group). Mean cortisol was found to be paradoxically higher in the CIRCI group (1341.6 ± 1112.5 vs. 976.0 ± 798.7 nmol/L, *p* = 0.24). ROC analysis demonstrated poor diagnostic performance (AUC 0.61, 95% CI: 0.44–0.78). International guideline cutoff of <276 nmol/L showed 0% sensitivity in identifying CIRCI. Multiple thresholds yielded negative Youden indices, indicating performance of cortisol levels being worse than a random chance. DCA demonstrated zero net benefit for cortisol-guided therapy across all threshold probabilities when compared to clincial practice strategies. Only clinical factors predicted corticosteroid initiation: high vasopressor requirements (OR 3.2, 95% CI: 1.1–9.4, *p* = 0.03) and persistent shock >48 h (OR 2.8, 95% CI: 1.0–7.9, *p* = 0.05). Cortisol level had no predictive value (OR 1.0, 95% CI: 0.9–1.0, *p* = 0.89). **Conclusions:** In this cohort, baseline total cortisol demonstrated poor diagnostic accuracy and lacked clinical utility for guiding corticosteroid therapy in patients with sepsis. Our findings reinforce the importance of clinical judgement over biochemical testing in identifying patients with septic shock requiring corticosteroid therapy, in line with the recent international guidelines.

## 1. Introduction

Critical illness-related corticosteroid insufficiency (CIRCI) is defined as an inadequate glucocorticoid response relative to illness severity, which often manifests as refractory hypotension in patients with septic shock [1]. Despite its recognition as a significant cause of morbidity and mortality, the diagnosis of CIRIC remains a contentious area in critical care medicine, primarily due to the absence of a diagnostic gold standard [2]. This diagnostic uncertainty stems from complex pathophysiology that fundamentally undermines the reliability of simple biochemical testing. The traditional aproach of using static cortisol measurements to diagnose adrenal insufficiency, while appropriate for outpatient endocrinology decision, can become fundamentally flawed in a critical care setting. Multiple physiological derangements occur simultaneously during sepsis: a massive inflammatory cytokine release alters hypothalamic–pituitary–adrenal (HPA) axis sensitivity, hepatic synthesis of cortisol-binding proteins fluctuates dramatically, and peripheral tissue glucocorticoid receptor expression and sensitivity change unpredictably [3]. Research has shown that cortisol half-life can increase from 90 min in healthy individuals to over 360 min in patients with critical illness, primarily due to suppressed activity of alpha-ring reductases (5α- and 5β-reductase) and 11 beta-hydroxysteroid dehydrogenase type 2 in the liver and kidney [4]. This impaired clearance, caused by suppressed cortisol-metabolizing enzyme activity in liver and kidneys, leads to accumulation of plasma cortisol and provides mechanistic explanation for frequent “ACTH–cortisol dissociation”, which is seen in patients with critical illness [5].

Furthermore, corticosteroid-binding globulin (CBG) concentration and integrity are dynamically regulated during sepsis. CBG levels decrease proportionally to illness severity through multiple mechanisms: reduced hepatic synthesis, increased vascular permeability leading to extravascular leak, and specific proteolytic cleavage by neutrophil elastase at inflammation sites. This elastase-mediated cleavage serves an evolutionary purpose, facilitating targeted cortisol delivery to inflamed tissues, but this makes the total cortisol measurements even less reliable [6]. The complex interplay of reduced metabolic clearance, altered protein binding, and potential tissue-level glucocorticoid resistance creates a profound disconnect between single plasma total cortisol measurements and true glucocorticoid bioactivity at the cellular level [7,8].

The diagnostic challenge of CIRCI is further complicated by emerging evidence of tissue-specific glucocorticoid resistance in sepsis. Even when circulating cortisol levels are elevated, cellular response may be inadequate due to decreased glucocorticoid receptor expression, altered receptor translocation, or impaired receptor–DNA binding [9]. This phenomenon explains why some patients with apparent hypercortisolemia still exhibit clinical features suggesting glucocorticoid insufficiency. The 2021 Surviving Sepsis Campaign and 2024 Society of Critical Care Medicine (SCCM) updates have fundamentally changed their approach, moving away from biochemical thresholds that dominated previous iterations. They now recommend corticosteroid initiation based primarily on clinical criteria, specifically the presence of septic shock requiring ongoing vasopressor support despite adequate fluid resuscitation [10,11]. This paradigm shift acknowledges that treatment decisions should be driven by clinical indicators like refractory shock rather than arbitrary, potentially misleading laboratory values that fail to capture the complex glucocorticoid physiology in patients with critical illness.

Population-specific variations in HPA axis function have been documented across different ethnic groups, influenced by genetic polymorphisms in genes encoding steroid-metabolizing enzymes, glucocorticoid receptors, and binding proteins [12]. Middle Eastern populations, in particular, may exhibit distinct cortisol metabolism patterns due to unique genetic backgrounds and environmental factors including chronic heat exposure, dietary patterns, and prevalence of metabolic syndrome [13]. To date, no systematic data on expected cortisol levels during septic shock have been published for Middle Eastern populations, creating a critical knowledge gap for clinicians treating increasingly diverse patient populations globally.

Therefore, this study aimed to evaluate the utility of baseline total cortisol measurement in patients with septic shock in the United Arab Emirates. Our objectives were threefold: first, to assess the diagnostic performance of various cortisol thresholds in predicting the clinical decision to administer corticosteroids; second, to determine the clinical utility of a cortisol-guided strategy using decision curve analysis; and third, to identify the clinical and biochemical factors that are independently associated with corticosteroid administration in real-world practice.

## 2. Methods

### 2.1. Study Design and Setting

This retrospective, single-center observational study was conducted at a 470-bedded tertiary care hospital in Al Ain, United Arab Emirates. The Regional Research Ethics Committee approved the study on 24 November 2022, granting an informed consent waiver due to the retrospective, de-identified data analysis nature. This manuscript was prepared according to Strengthening the Reporting of Observational Studies in Epidemiology (STROBE) guidelines [14].

### 2.2. Study Population and Definitions

Electronic medical records of all adult patients (age ≥ 18 years) admitted to the intensive care unit between 1 June 2012 and 1 June 2022 were screened. Patients with International Classification of Diseases, 10th Revision (ICD-10) code R65.20 (severe sepsis) or R65.21 (septic shock) who required high-dose vasopressor support within 48 hours (h) of presentation and had serum cortisol level measured during admission were eligible for inclusion. High-dose vasopressor support was defined as a norepinephrine requirement of >0.5 μg/kg/min or the use of multiple vasopressor agents. While no universal consensus exists for defining ‘high-dose’ vasopressors, this threshold was chosen as it represents a clinically significant level of vasopressor dependency often associated with refractory shock and consideration for adjunctive therapies [15]. Exclusion criteria included other shock etiologies (e.g., cardiogenic, hemorrhagic), known adrenal insufficiency, chronic corticosteroid use (prednisolone equivalent ≥7.5 mg for ≥6 weeks), or corticosteroid exposure within 24 h prior to baseline cortisol measurement.

The primary outcome for this study was the clinical decision to administer intravenous hydrocortisone for a minimum of 24 h. In the absence of a biological gold standard for CIRCI, this outcome serves as a pragmatic proxy for the clinician’s judgment that a patient has refractory shock and is likely to benefit from corticosteroid administration. Therefore, this study evaluates the factors, both clinical and biochemical, that predict this real-world treatment decision, rather than diagnosing an independent biological entity. Inherent limitations of this approach are addressed in the Discussion. Patients receiving hydrocortisone were assigned to the “CIRCI group,” while those not receiving it were assigned to the “non-CIRCI group.”

### 2.3. Data Collection and Assays

Data were extracted from hospital electronic medical records, including patient demographics, comorbidities, sepsis source, clinical parameters (e.g., vasopressor requirements, length of stay), laboratory values, and mortality outcomes. Serum cortisol was quantified using a Roche Cobas Generation-II electrochemiluminescence competitive immunoassay (Elecsys^®^ Cortisol II, Roche Diagnostics, Basel, Switzerland) with high specificity (<13% cross-reactivity with other steroids) and excellent precision (intra-assay coefficient of variation 1.7%, inter-assay 2.8%) [16].

### 2.4. Statistical Analysis

Data distribution was assessed using the Shapiro–Wilk test. Continuous variables are presented as means ± standard deviations (SDs) or medians with interquartile ranges (IQRs), compared using the Student’s *t*-test or Mann–Whitney U test, respectively. Categorical variables are expressed as frequencies and percentages, compared using the chi-square or Fisher’s exact test. To supplement inferential testing, effect sizes were calculated to quantify the magnitude of the differences between groups. Cohen’s d was used for continuous variables, and Cramér’s V was used for categorical variables.

### 2.5. Diagnostic Performance Analysis

Receiver Operating Characteristic (ROC) curve analysis assessed the discriminatory ability of baseline cortisol levels to predict the clinical decision to treat. The area under the curve (AUC) was calculated as global test performance measure. For various cortisol thresholds, sensitivity, specificity, positive predictive value (PPV), and negative predictive value (NPV) were determined. Youden index (J = sensitivity + specificity − 1) evaluated overall diagnostic effectiveness of each threshold. Youden index ≤ 0 indicates no diagnostic value, while negative index signifies performance worse than random chance [17].

### 2.6. Clinical Utility Analysis

Decision Curve Analysis (DCA) evaluated the clinical usefulness of the cortisol model beyond simple accuracy metrics [18]. DCA assesses whether using a prediction model to guide clinical decisions provides greater “net benefit” than default clinical strategies. Threshold probability (Pt) represents disease probability at which a clinician would opt for treatment. Net benefit quantifies clinical value by weighing true positives (benefits) against false positives (harms) relative to the chosen threshold probability [19]. The decision curve for the cortisol model was compared against two default strategies: “Treat All” (initiating hydrocortisone in all patients with refractory shock) and “Treat None.” Model is considered clinically useful only if its decision curve lies above both default strategies across clinically reasonable threshold probabilities range.

### 2.7. Predictor Analysis

A multivariate logistic regression model identified clinical and biochemical factors independently associated with corticosteroid therapy initiation. Variables with *p*-value < 0.20 in bivariate analysis were considered for inclusion. Two-tailed *p*-value < 0.05 was considered statistically significant for all analyses. Statistical analyses were performed using SPSS version 29.0 (IBM Corp., Armonk, NY, USA).

## 3. Results

### 3.1. Patient Characteristics and Clinical Outcomes

**Figure 1:**
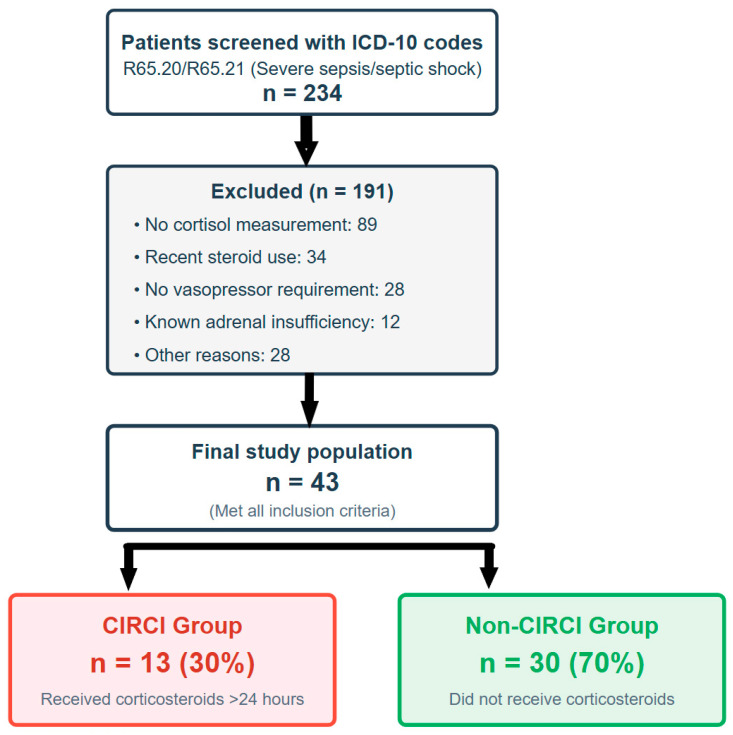
Patient selection flowchart following STROBE guidelines. Flowchart of patient selection from 234 screened patients with severe sepsis/septic shock (June 2012–June 2022). Patients clinically identified as having critical illness-related corticosteroid insufficiency (CIRCI) were in the CIRCI group and received corticosteroids for >24 h, while the non-CIRCI group did not.

Of 234 patients screened with relevant ICD-10 codes for severe sepsis or septic shock, 43 met the final inclusion criteria. See Figure 1 for a patient flow diagram as per STROBE guidelines.

The cohort was divided into the CIRCI group (*n* = 13), who received hydrocortisone for >24 h, and the non-CIRCI group (*n* = 30). The primary sepsis source was pneumonia (37.2%), followed by urinary tract (23.3%) and abdominal infections (18.6%). A causative organism was not identified in 41.9% of cases. The patient population was severely ill, with an overall in-hospital mortality rate of 69.8% (30/43). The median ICU length of stay was 14 days (IQR: 8–21). All patients required vasopressor support, with 41.9% requiring multiple agents. Baseline characteristics and outcomes were similar between groups, except that pneumonia was a significantly more common sepsis source in the CIRCI group (61.5% vs. 26.7%, *p* = 0.02). See Table 1 for details.

### 3.2. Cortisol Level Analysis and Diagnostic Performance

Surprisingly, a paradoxical trend was observed in cortisol levels. Mean baseline cortisol in the CIRCI group was higher than in the non-CIRCI group (1341.6 ± 1112.5 nmol/L vs. 976.0 ± 798.7 nmol/L), although this difference did not reach statistical significance (*p* = 0.24). The overall cortisol value range was extremely wide, spanning 89 to 3311 nmol/L.

**Figure 2:**
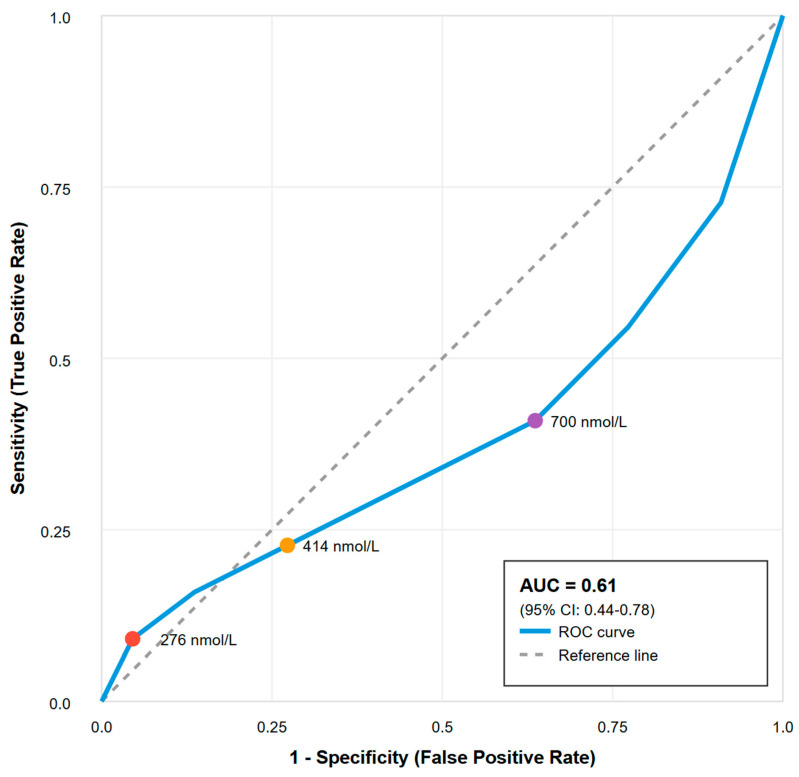
Receiver operating characteristic curve for prediction of critical illness-related corticosteroid insufficiency. Receiver Operating Characteristic curve analysis evaluating the diagnostic performance of baseline cortisol levels for predicting clinically defined critical illness-related corticosteroid insufficiency (CIRCI). Key cut-off values for cortisol (276, 414, and 700 nmol/L) are marked on the curve. Area under the curve (AUC) = 0.61 (95% CI: 0.44–0.78), indicating poor discriminatory ability. The diagonal grey dashed line represents the “Line of No Discrimination” or reference line, which shows the expected performance of a random classifier (AUC = 0.5) that has no diagnostic value whatsoever.

ROC curve analysis evaluating the baseline cortisol ability to predict clinical decisions to treat yielded an AUC of 0.61 (95% CI: 0.44–0.78), indicating poor discriminatory ability, barely better than chance. See Figure 2.

Critically, all tested cortisol cutoffs demonstrated inadequate diagnostic performance. The international guideline cutoff of <276 nmol/L had 0% sensitivity for identifying patients ultimately treated with corticosteroids. Furthermore, several thresholds, including 414 nmol/L and 700 nmol/L, yielded negative Youden indices (−0.036 and −0.182, respectively). This finding indicates that using these cutoffs to guide therapy would result in more misclassifications than a random coin toss, making them actively misleading. The best-performing cutoff, 350 nmol/L, achieved a Youden index of only 0.054, a value negligibly different from zero. See Table 2 for details.

### 3.3. Clinical Utility of Cortisol Measurement

**Figure 3:**
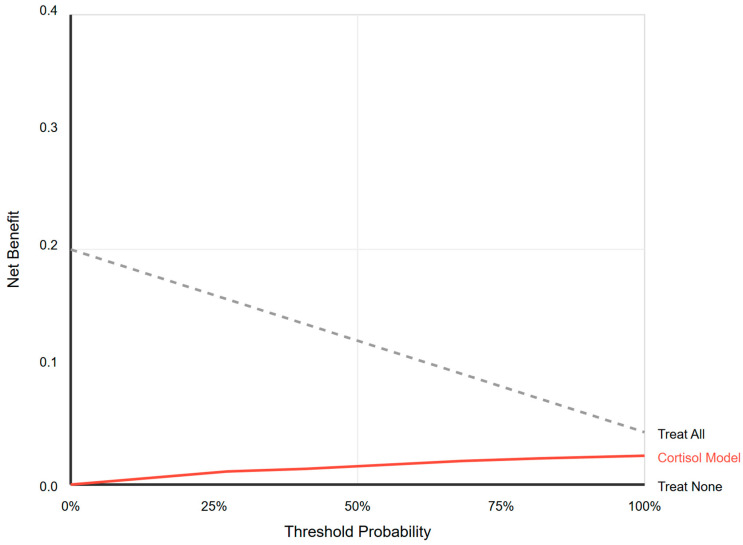
Decision curve analysis. Net benefit analysis comparing cortisol-guided treatment versus treating all or none. The cortisol model provides no clinical utility at any threshold probability.

Decision curve analysis determined if using baseline cortisol to guide treatment decisions offered any clinical advantage over default strategies. The decision curve for the cortisol model failed to show net benefit greater than that of the default strategies of “Treat All” or “Treat None” across the entire plausible threshold probability range (0–100%). See Figure 3.

For any given threshold at which a clinician might consider starting hydrocortisone, relying on cortisol levels provided no additional clinical value and was inferior to the strategy of simply treating all patients with refractory shock based on clinical judgment alone. This demonstrates a complete lack of clinical utility for tests in this setting.

### 3.4. Predictors of Corticosteroid Initiation

Multivariate logistic regression analysis confirmed that the decision to initiate corticosteroid therapy was driven exclusively by clinical factors indicative of refractory shock. High vasopressor requirements were a significant predictor (OR 3.2; 95% CI: 1.1–9.4; *p* = 0.03). Persistent shock lasting more than 48 h also significantly predicted treatment initiation (OR 2.8; 95% CI: 1.0–7.9; *p* = 0.05). In stark contrast, baseline cortisol levels showed no association whatsoever with the decision to treat (OR 1.0 per 100 nmol/L increase; 95% CI: 0.9–1.0; *p* = 0.89), confirming that clinicians appropriately disregarded this biochemical marker in their decision-making process. See Table 3 for details.

## 4. Discussion

This study, the first to evaluate cortisol dynamics in septic shock in a UAE population, provides compelling evidence for the poor diagnostic performance of baseline total cortisol as a CIRCI marker. The analysis demonstrates that cortisol lacks not only diagnostic accuracy, evidenced by poor ROC performance and negative Youden indices, but also clinical utility, shown by the complete absence of net benefit in decision curve analysis. These findings suggest that the long-standing pursuit of a diagnostic cortisol threshold for CIRCI may be fundamentally flawed.

### 4.1. Pathophysiological Basis for Diagnostic Failure

The lack of clinical utility of total cortisol as a diagnostic marker is not an anomaly but rather an expected consequence of significant pathophysiological HPA axis derangements during critical illness [13]. The paradoxical observation that patients who are clinically deemed to have CIRCI had higher mean cortisol levels aligns perfectly with the modern understanding of cortisol dynamics in critical illness. This apparent contradiction reflects multiple simultaneous physiological derangements that render static cortisol measurements meaningless.

First, the profound reduction in cortisol metabolism during critical illness fundamentally alters the interpretation of plasma cortisol levels. The normal cortisol half-life of approximately 90 min extends to over 6 h in critically ill patients [4]. This occurs through the suppression of multiple enzymatic pathways, including 5α-reductase and 5β-reductase, and their activity in the liver decreases by up to 80%, while 11β-hydroxysteroid dehydrogenase type-2 activity in the kidney is similarly suppressed [20]. The result is plasma cortisol accumulation that reflects impaired clearance rather than enhanced production. Our finding of elevated cortisol in critically ill patients requiring steroids likely represents this clearance failure rather than adequate adrenal response.

Second, the alterations in cortisol-binding proteins during critical illness can create additional interpretive challenges. Research has shown that while total cortisol may appear normal or even low due to hypoalbuminemia and reduced CBG, free cortisol levels can be markedly elevated [21]. The elastase-mediated cleavage of CBG at inflammatory sites, while serving the evolutionary purpose of enhancing local cortisol delivery, further disconnects total cortisol measurements from biological activity [22]. Given the retrospective nature of the study without any free cortisol measurements, we could only measure total cortisol. Our findings exhibit that total the cortisol values lack any diagnostic utility in this context.

Third, emerging evidence of tissue-specific glucocorticoid resistance provides the final explanation for our paradoxical findings. Current research reveals that glucocorticoid receptor expression and sensitivity vary dramatically among septic patients, with those exhibiting greatest resistance having paradoxically the highest cortisol levels [23]. This resistance occurs through multiple mechanisms: decreased receptor expression, impaired nuclear translocation, altered cofactor availability, and enhanced expression of the inhibitory β-isoform of the glucocorticoid receptor [24]. This explains why patients with the highest cortisol levels in our cohort were those who were clinically identified to have CIRCI and were started on corticosteroids. This study’s findings serve as real-world clinical confirmation of these established molecular mechanisms, bridging the gap between bench-level understanding and bedside clinical practice.

### 4.2. The Primacy of Clinical Judgment

Our multivariate analysis results validate treating clinicians’ actions. The exclusive reliance on clinical indicators such as high vasopressor requirements and persistent shock, while completely ignoring cortisol values, demonstrated appropriate clinical judgment that aligns with physiological understanding. These clinical markers directly reflect the problem corticosteroids are intended to address: hemodynamic instability from relative glucocorticoid insufficiency at the tissue level. This approach is further validated by the consistent findings from major randomized controlled trials. The ADRENAL trial (n = 3800), the largest study to date, found no mortality benefit at 90 days (27.9% vs. 28.8%, *p* = 0.50) but demonstrated faster shock reversal with hydrocortisone [25]. Similarly, the CORTICUS trial showed accelerated shock reversal without survival benefit [26]. Our identical mortality rates between groups (69.2% vs. 70.0%) provide real-world confirmation of these RCT findings in a Middle Eastern population. The message is clear: corticosteroids may help reverse shock faster but do not save lives, making clinical assessment of shock severity the only relevant factor for treatment decisions.

**Figure 4:**
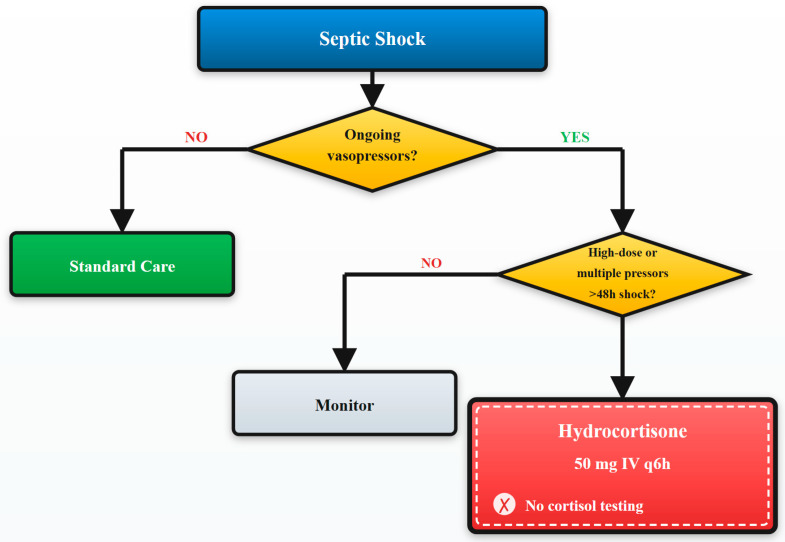
Clinical decision algorithm for management of patients with critical illness-related corticosteroid insufficiency. Evidence-based algorithm for management of patients with critical illness-related corticosteroid insufficiency (CIRCI). Clinical criteria guide treatment decisions without cortisol testing, based on vasopressor requirements and shock duration.

This study’s findings provide strong, real-world evidence supporting recent international guidelines’ shift away from biochemical triggers toward management strategies guided exclusively by clinical assessment [10,11]. See Figure 4 for details.

The 2024 SCCM focused update specifically states that cortisol testing should not be used to identify patients with septic shock who should receive corticosteroids, a recommendation our data strongly support [11].

### 4.3. Regional Considerations and Global Implications

While our population exhibited elevated baseline cortisol levels compared to Western cohorts, this finding reinforces rather than contradicts the diagnostic futility of cortisol testing. The wide variability within our cohort (89–3311 nmol/L) and complete overlap between CIRCI and non-CIRCI groups make any population-specific threshold impossible to define. Whether these elevations reflect genetic polymorphisms in cortisol-metabolizing enzymes, chronic heat stress adaptations, or higher prevalence of metabolic syndrome in our population is academically interesting but clinically irrelevant [27].

Our findings align with emerging international evidence questioning cortisol utility across diverse populations. Recent studies from Indonesia and COVID-19 cohorts also reveal poor diagnostic performance of cortisol testing, though none have shown the significant lack of clinical utility that we document [28,29]. The negative Youden indices we report represent a new low in diagnostic test performance, indicating that using these thresholds would literally be worse than flipping a coin.

### 4.4. Strengths and Limitations

This study’s main strength is its unique position as the first “real-world” cortisol level evaluation in septic shock within a Middle Eastern population. While the small sample size requires caution, cortisol demonstrated significantly poor performance, with an area under the ROC curve being only 0.61, and several key thresholds yielded negative Youden indices, suggesting that the conclusion of poor performance is robust despite the limited sample. Additional strengths include the comprehensive statistical approach combining ROC analysis, Youden indices, and Decision Curve Analysis, a methodological rigor rarely seen in the CIRCI literature. The 10-year study period provides robust data despite the relatively rare condition.

However, several important limitations must be acknowledged. The primary limitation of this study is its retrospective design and the use of corticosteroid administration as the primary outcome. This approach does not allow for a true assessment of diagnostic accuracy against an independent gold standard for CIRCI, as such a standard does not exist. However, this design provides a unique strength: it allows for a pragmatic evaluation of the factors that influence real-world clinical decision-making. Our study effectively answers the question, “Do cortisol levels or clinical factors predict the decision to prescribe steroids in septic shock?” and, in doing so, assesses the alignment of this biochemical marker with expert clinical judgment.

Second, the modest sample size (N = 43) limits statistical power and increases the risk of overfitting in the multivariate model. To mitigate this, we constructed a parsimonious model by strictly limiting the number of predictors; however, the potential for overfitting cannot be entirely excluded. However, even this modest sample size yielded significant results. When a diagnostic test performs worse than random chance, increasing sample size would only confirm its uselessness more precisely.

Thirdly, the decision to initiate corticosteroids is complex and may have been influenced by unmeasured confounders or individual clinician practices not captured in the electronic medical record.

Finally, this study did not include dynamic testing with cosyntropin stimulation or direct free cortisol measurement. While these are recognized limitations, the poor diagnostic performance of cortisol makes it highly unlikely that these more complex and expensive tests would prove useful. If the simpler test has zero utility, adding complexity can rarely help. Moreover, neither cosyntropin stimulation testing nor free cortisol measurement has been proven to improve patient-important outcomes in critical illness [30,31].

### 4.5. Implications for Clinical Practice and Future Research

Our findings have direct implications for clinical practice and resource stewardship. We recommend that the clinicians discontinue the routine use of baseline total cortisol testing to guide corticosteroid therapy in septic shock. Instead, the decision to treat should be based on a clinical assessment of shock severity, primarily the persistence of hypotension despite fluid resuscitation and the requirement for high-dose vasopressors. This approach not only aligns with the latest international guidelines but also simplifies bedside decision-making, avoids the confusion of misleading test results, and eliminates the costs associated with a low-value diagnostic practice. Future research should move away from the search for a diagnostic cortisol threshold and instead concentrate on identifying clinical phenotypes of steroid responsiveness, sepsis endotypes, and development of future biomarkers of tissue-level glucocorticoid resistance.

## 5. Conclusions

This study, conducted in a UAE cohort of septic shock patients, provides evidence that baseline total cortisol has poor diagnostic performance and limited clinical utility as a marker for CIRCI. All tested biochemical thresholds performed poorly, with several performing worse than random chance, and Decision Curve Analysis confirmed the lack of any clinical utility. The paradoxical cortisol elevation in the most severely ill patients further undermines the conceptual basis of a simple biochemical diagnosis. Our findings suggest that clinical judgment, based on hemodynamic parameters, such as high vasopressor requirements and shock duration, should remain the primary guide for initiating corticosteroid therapy. The pursuit of a single diagnostic biochemical threshold for CIRCI may be a futile exercise given the complex underlying pathophysiology of septic shock.

## Figures and Tables

**Table 1:** Baseline characteristics of patients.

Characteristic	Total (N = 43)	CIRCI Group (*n* = 13)	Non-CIRCI Group (*n* = 30)	*p*-Value	Effect Size
Clinical Variables					
Age (years), mean ± SD	62.3 ± 15.8	64.1 ± 14.2	61.5 ± 16.5	0.62	0.16 (d)
Male sex, *n* (%)	26 (60.5)	8 (61.5)	18 (60.0)	0.92	0.01 (V)
ICU LOS (days), median (IQR)	14 (8–21)	16 (10–24)	13 (7–19)	0.31	
Mortality, *n* (%)	30 (69.8)	9 (69.2)	21 (70.0)	0.95	0.01 (V)
Sepsis source					
Pneumonia, *n* (%)	16 (37.2)	8 (61.5)	8 (26.7)	0.02	0.35 (V)
Urinary tract, *n* (%)	10 (23.3)	2 (15.4)	8 (26.7)	0.42	0.17 (V)
Abdominal, *n* (%)	8 (18.6)	2 (15.4)	6 (20.0)	0.71	0.08 (V)
Vasopressor Support					
Multiple vasopressors, *n* (%)	18 (41.9)	8 (61.5)	10 (33.3)	0.08	0.28 (V)
* High-dose vasopressors, *n* (%)	15 (34.9)	7 (53.8)	8 (26.7)	0.08	0.27 (V)
Cortisol Levels (nmol/L)					
Mean ± SD	1081.4 ± 880.1	1341.6 ± 1112.5	976.0 ± 798.7	0.24	0.41 (d)
Median (IQR)	700 (456–1500)	998 (529–1731)	635.5 (405–1392)	0.28	
Metabolic Parameters					
Glucose (mmol/L), mean ± SD	7.2 ± 2.4	8.12 ± 2.8	6.8 ± 2.1	0.09	0.57 (d)
Hyponatremia (<135 mmol/L), *n* (%)	12 (27.9)	5 (38.5)	7 (23.3)	0.31	0.18 (V)
Hyperkalemia (>5.0 mmol/L), *n* (%)	8 (18.6)	3 (23.1)	5 (16.7)	0.62	0.09 (V)

Abbreviations: CIRCI, critical illness-related corticosteroid insufficiency; ICU, intensive care unit; LOS, length of stay; IQR, interquartile range; SD, standard deviation. Effect sizes are reported as Cohen’s d (d) for continuous variables (small ≥0.2, medium ≥0.5, large ≥0.8) and Cramér’s V (V) for categorical variables (small ≥0.1, medium ≥0.3, large ≥0.5). * Norepinephrine > 0.5 μg/kg/min or equivalent.

**Table 2:** Diagnostic performance of cortisol cutoff values to predict critical illness-related corticosteroid insufficiency.

Cutoff (nmol/L)	Sensitivity (%)	Specificity (%)	PPV (%)	NPV (%)	Youden Index	Interpretation
276	0.0	96.7	0.0	69.8	**−0.033**	**No diagnostic value**
300	7.7	96.7	50.0	70.7	0.044	Negligible value
350	15.4	90.0	40.0	71.1	0.054	Best but still poor
400	23.1	80.0	30.0	72.7	0.031	Poor performance
414	23.1	73.3	27.3	68.8	**−0.036**	**No diagnostic value**
500	30.8	70.0	28.6	72.4	0.008	Negligible value
700	38.5	43.3	20.0	65.0	**−0.182**	**Worse than random**
1000	53.8	36.7	23.1	68.8	**−0.095**	**Worse than random**

Abbreviations: PPV, positive predictive value; NPV, negative predictive value. Note: The Youden index (J = Sensitivity + Specificity − 1) measures a test’s ability to balance sensitivity and specificity, ranging from −1 to +1. A value of J ≤ 0 indicates that the test has no diagnostic utility at that threshold. A negative index signifies that the test performs worse than random chance, resulting in more misclassifications than correct classifications. Bold text emphasizes the worst performing categories, with no diagnostic value (for cutoffs with Youden index ≤0)

**Table 3:** Multivariate analysis of factors associated with corticosteroid treatment.

Variable	Odds Ratio	95% CI	*p*-Value	Clinical Significance
Clinical Factors				
* High vasopressor requirements	**3.2**	1.1–9.4	**0.03**	**Significant predictor**
Prolonged shock (>48 h)	**2.8**	1.0–7.9	**0.05**	**Significant predictor**
Pneumonia source	2.1	0.7–6.3	0.18	Trend only
**+** Multiple organ failure	1.8	0.6–5.2	0.29	Not significant
Laboratory Factors				
**++** Baseline cortisol level	**1.0**	0.999–1.001	**0.89**	**No predictive value**
Hyponatremia (<135 mmol/L)	1.6	0.5–4.8	0.42	Not significant
Hyperkalemia (>5.0 mmol/L)	1.4	0.4–4.5	0.58	Not significant
Acute kidney injury	1.2	0.3–4.6	0.78	Not significant

Abbreviations: CI, confidence interval. * Multiple vasopressors or norepinephrine >0.5 μg/kg/min. **+** ≥3 organ systems involved. **++** Per 100 nmol/L increase. Note: Cortisol level has NO association with treatment decisions (OR 1.0, *p* = 0.89), while clinical severity markers are the only significant predictors. Bold indicates variables with significant *p*-value in predicitng the clinical condition in comparison with baseline cortiosl level.

## Data Availability

Data are available from corresponding author upon reasonable request.
